# Gut microbial IgA coating in infants with traditional farming lifestyle and urban infants with allergic outcomes

**DOI:** 10.3389/fimmu.2026.1793302

**Published:** 2026-04-20

**Authors:** Kevin Bu, Tyler Scherzi, Adam Cantor, Andy A. Teng, Jozelyn V. Pablo, Adam D. Shandling, Arlo Z. Randall, Erin Davis, Courtney Jackson, R. John Looney, Joseph J. Campo, Antti Seppo, Kirsi M. Järvinen, Jose C. Clemente

**Affiliations:** 1 Department of Genetics and Genomic Sciences, Icahn School of Medicine at Mount Sinai, New York, New York, NY, United States; 2 Division of Allergy and Immunology and Center for Food Allergy, Department of Pediatrics, University of Rochester School of Medicine and Dentistry and Golisano Children’s Hospital, Rochester, NY, United States; 3 Antigen Discovery, Inc., Irvine, CA, United States; 4 Division of Allergy, Immunology and Rheumatology, Department of Medicine, University of Rochester School of Medicine and Dentistry, Rochester, NY, United States

**Keywords:** Atopic dermatitis, early life, food allergy, IgA, microbiome

## Abstract

**Background:**

The sharp increase in prevalence of atopic disease suggests a role for environmental factors, such as the microbiome. Here, we study the impact of immunoglobulin A (IgA) coating of gut bacteria in infancy on allergic outcomes in two distinct populations: (1) an urbanized cohort of Rochester infants (ROC) enriched for allergies (prevalence of 40%) and (2) infants from a traditional, agrarian Old Order Mennonite (OOM) community with a low prevalence of allergies (less than 2%).

**Methods:**

We performed immunoglobulin A sequencing (IgA-SEQ) on stool samples collected at an average of 6 months of life to assess gut microbiome IgA coating levels in 9 OOM and 21 ROC infants. Atopic outcomes were diagnosed throughout the first 2 years; 10 of the ROC infants were diagnosed with atopic dermatitis and/or food allergy, while none of the OOM infants were allergic. We also assessed human milk IgA-binding of taxa-derived protein antigens, as well as IgA binding to live bacterial cell cultures.

**Results:**

Gut microbiome composition was dominated by *Bifidobacterium*, followed by *Ruminococcus*, Enterobacteriaceae, and *Blautia*. Higher IgA coating of *P. melaninogenica* and Pasteurellaceae were associated with allergic outcomes and higher coating of *R. gnavus* was observed in non-allergic infants. IgA coating levels of *Atopobium*, *Bifidobacterium*, and *Coprococcus* were positively associated with infant age, and coating levels of *Corynebacterium* associated negatively with infant age. In non-allergic infants, IgA coating of *Clostridium* was decreased, while in allergic infants, IgA coating of *Corynebacterium* was decreased. Furthermore, breastfeeding was associated with higher levels of fecal IgA in infancy, and IgA-binding capacity to *B. infantis*, a keystone infant commensal, was subsequently assessed using *in vitro* experiments. Compared to the ROC cohort, milk from OOM mothers exhibited a higher level of IgA response to *B. infantis* and several other commensals. Surprisingly, IgA-binding to *B. infantis* was partially mediated by Fab-independent interactions through binding to glycosylated regions of immunoglobulins.

**Conclusion:**

Differential gut microbial IgA coating may play a role in development of allergic diseases in infancy. Human milk from communities with low rates of allergic diseases exhibit higher IgA responses to infant commensals, including *B. infantis*.

## Introduction

Allergic diseases remain an important clinical problem, as we lack approaches to cure them, and prevention strategies are limited. Approximately 30% of adults and 27% of infants are thought to have some form of an allergic disease (1, 2). The rapid increase in prevalence over the last few decades (3) suggests that, despite known genetic risk factors (4), environmental triggers likely play an important role in their pathogenesis. These triggers are thought to be particularly relevant during early life, when the immune system is developing to recognize and distinguish safe substances from potentially pathogenic agents (5).

Among these environmental factors, the microbiome can modulate atopic risk and its resolution during early life, as we and others have shown (6–10). While mechanistically informative, studies on the microbiome in animal models of allergies do not necessarily translate to conditions found in wild mammals or to human biology (11, 12). On the other hand, human studies recruiting participants from the general population are impacted by practices that alter the microbiome, such as delivery mode, antibiotics, or diet (5, 13). Studying populations that maintain traditional lifestyles and exhibit a low prevalence of immune diseases thus offers an opportunity to understand protective microbial factors (14, 15). Old Order Mennonites are groups of Swiss German heritage that, despite living in industrialized countries, conserve an agrarian lifestyle with limited use of modern technologies. They also maintain practices that are thought to support early life microbiome development, including vaginal deliveries, extended breastfeeding, and minimal use of antibiotics. Importantly, rates of allergic disease are notably lower in these communities compared to the general population (16), suggesting that their traditional lifestyle facilitates the development of a microbiome that potentially leads to a more tolerogenic state (7).

Deciphering how microbial colonization in early life induces immune responses from the host requires an understanding of immunoglobulin A (IgA), the major immunoglobulin in the gut. Newborns are unable to produce sufficient amounts of IgA during the first months of life and obtain it primarily from their mothers through breastfeeding. IgA is found in high concentrations in human milk (17, 18), and infants who are breastfed have higher fecal IgA concentration that increases with exclusivity of breastfeeding (19). The levels of IgA in human milk can however vary between mothers, with lactation stage, and depending on environmental factors (17). Self-production by the infant is generally thought to start from 2 to 4 weeks of age (20), and recent work has characterized changes in IgA levels over the first year of life, although not specifically in the setting of allergies (21).

While IgA has been traditionally studied in the context of recognition and clearance of enteric pathogens (22), IgA binding can also promote bacterial epithelial adherence, biofilm formation, and mucosal colonization, both in adults and infants (23–27). For example, IgA coating of *Lacticaseibacillus rhamnosus* and *Bifidobacterium lactis*, two organisms commonly utilized as probiotics (28), caused increased bacterial adhesion, improved barrier function, and nuclear translocation of NF-kB. These observations highlight potential mechanisms by which IgA coating of commensal bacteria can induce specific host responses (29). Technologies to distinguish IgA coating levels of different microbes, such as IgA-SEQ, are thus critical to understand host-microbe interactions and their relationships with health outcomes (30). Bacteria with high IgA coating can trigger proinflammatory T_H_17 responses (31) or worsen disease progression in inflammatory bowel disease (32), but also protect against colitis and increase efficacy of fecal microbiota transplantation in ulcerative colitis (33). Taken together, these examples demonstrate that IgA coating can identify bacteria that are pathogenic (30, 31) or protective (25, 33), suggesting complementary mechanisms of immunomodulation (30, 32).

However, the precise mechanisms by which IgA interacts with bacteria to shape early life colonization and allergic risk remain poorly understood, particularly in the context of populations with protective lifestyle factors. Here, we analyzed a cohort of infants from an Old Order Mennonite community with a low prevalence of allergies and matched controls from Rochester at high risk for allergies, as we have previously described (18). We measured fecal IgA coating of bacteria, analyzed its association with population of origin and allergic outcomes, and explored potential relationships between coating levels and infant age. Additionally, utilizing a high-density microarray, we probed human milk from mothers in this cohort for IgA reactivity towards commensal bacterial antigens and determined mechanistic components of IgA binding to *Bifidobacterium* and other bacteria in early infancy. By combining IgA-SEQ profiling of microbial communities in infants from two distinct populations with high-throughput microarray characterization of milk IgA-binding capacity, our study provides novel insights into potential mechanisms associated with allergic outcomes.

## Results

### Study design and cohort description

**Figure 1:**
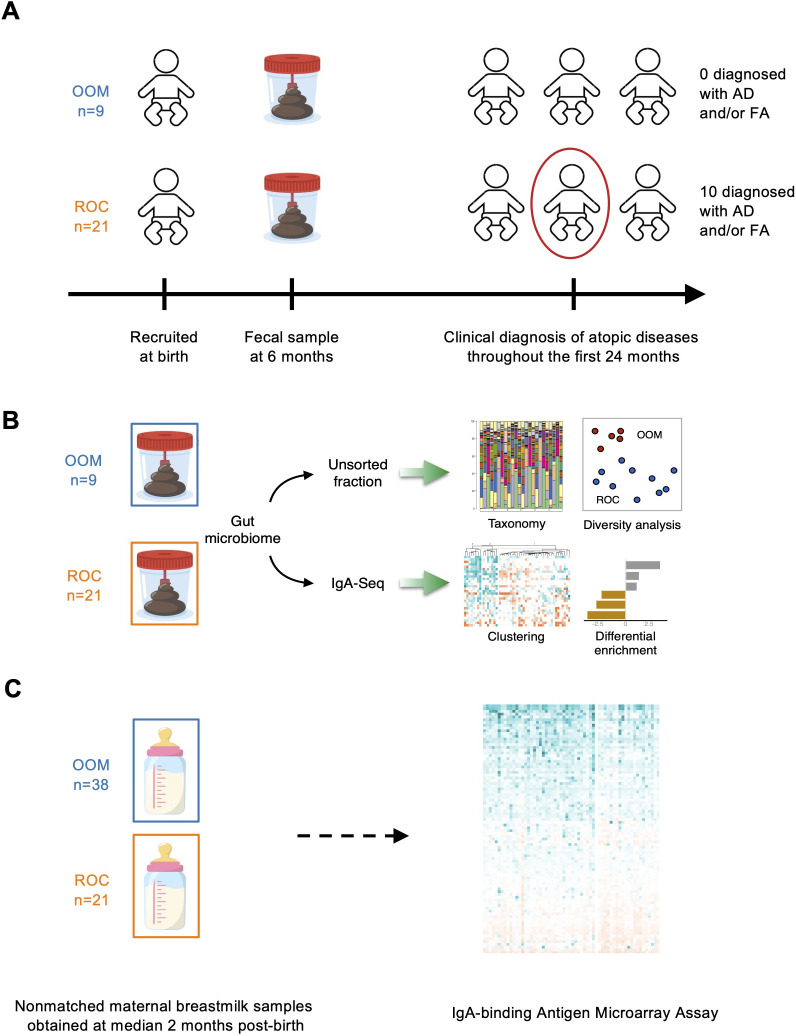
Study design. **(A)** Cohort design, sample acquisition, and timeline of assessment of allergies. **(B)** IgA-SEQ microbiome analysis in relation to allergic outcomes. **(C)** Human milk IgA binding on protein array.

Fecal samples utilized for IgA-SEQ were selected among a larger birth cohort of infants previously described (34) that represents infants from agrarian and urban lifestyles with well-characterized allergic outcomes. Our protocol was approved by the Institutional Review Board of the University of Rochester Medical Center (RSRB52971) and all subjects provided written consent prior to enrollment to the study. We included 9 Old Order Mennonite (OOM) infants from Western New York who are at low risk for allergic diseases due to their lifestyle and 21 infants born to atopic families in suburban/urban Rochester, NY (ROC). Based on sample availability and to achieve a balanced study design, we selected approximately equal numbers of OOM, ROC allergic, and ROC non-allergic infants. Allergic outcomes (atopic dermatitis and/or food allergy) were diagnosed by a physician throughout the first 24 months of life based on both the clinical presentation of an IgE-mediated food allergy and positive skin prick testing and/or specific IgE as detailed in prior work (**Figure 1A**) (34). **Table 1** describes demographics and characteristics of infants whose stool samples were assessed in this study. **Supplementary Tables 1**, **2** describe the cohort with respect to allergic outcomes in both populations and in ROC infants alone. Stool samples were collected from diapers of infants throughout the first year of life; samples utilized here were from 6 months of age visit (**Figure 1B**). In addition to taxonomic profiling and IgA-SEQ, we performed protein-binding microarray assay on human milk derived from available samples collected at an average of 2 months of lactation from OOM and ROC mothers as part of our previously published cross-sectional study of mother-infant pairs in the same OOM and ROC populations (7). (**Figure 1C**). Samples were immediately frozen in home freezers and subsequently transported to the laboratory and stored in freezers at -80C until processed for further assays. See **Supplementary Materials** for a detailed description of the cohort and samples.

**Table 1:** Cohort demographics and allergic outcomes for samples used for IgA-SEQ.

Covariate	Rochester N=21		Old order mennonites N=9		p-value
Age (days, median ± s.d.)	182 ± 33		205 ± 36		0.023
Male/female	6/15		7/2		1.0
Vaginal/C-section	16/5		9/0		0.15
	Yes	No	Yes	No	
Antibiotics	5	16	1	8	0.64
Some breastfeeding	17	4	9	0	0.29
Cat in household	6	15	7	2	0.02
Dog in household	13	8	7	2	0.68
Any allergic outcomes	10	11	0	9	0.01
Food allergy*	6	15	0	9	0.15
Atopic dermatitis	10	11	0	9	0.01

Cell values indicate number of infants in each cohort corresponding to each covariate. Cumulative incidence of food allergy and atopic dermatitis diagnosed by 24 months of age. *Food allergy outcomes include three infants with egg and tree nuts, one with egg alone, one with fish and one with shellfish and banana. The number of allergic outcomes is not mutually exclusive; all six infants with food allergy also had atopic dermatitis. P-values indicate tests of association using Student’s T-Test for continuous variables and Fisher’s Exact Test for categorical variables.

### Unsorted microbial composition and diversity were not significantly different with respect to population or allergic outcome

**Figure 2:**
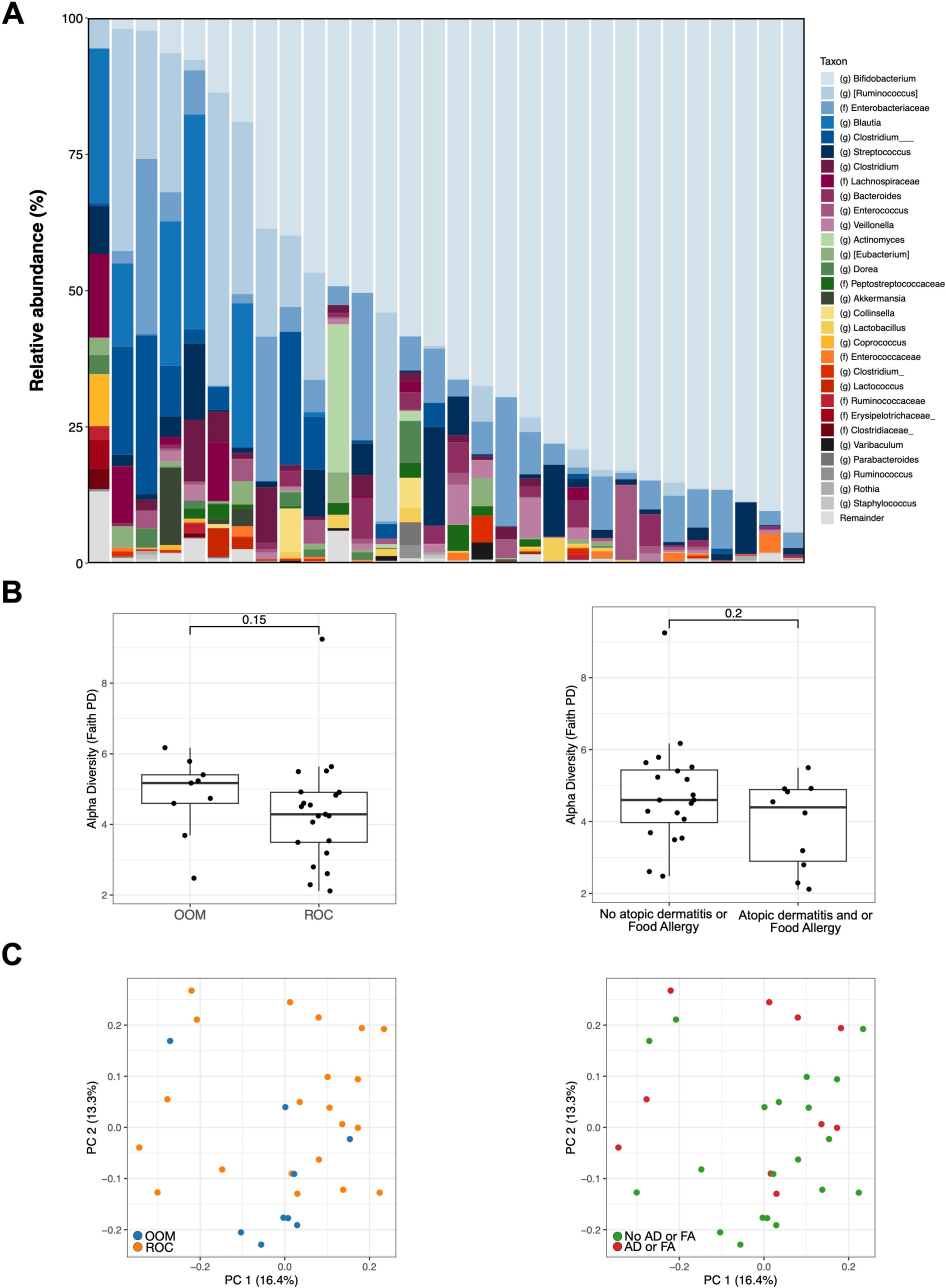
Unsorted microbiome composition and diversity. **(A)** Taxa summary plot per sample. **(B)** Alpha diversity with respect to population and allergic outcome. **(C)** PCoA plots with samples colored by population and allergic outcome.

We analyzed our data in terms of both overall pre-sorted abundances (i.e. from aliquots of the sample that had not been sorted and thus contain no information on IgA coating) and with respect to IgA coating (**Figure 1B**). Samples representing the unsorted fraction of the microbiome had a mean coverage of 48,820 ± 20,853 reads/sample, with a total of 495 amplicon sequence variants (ASVs). Microbiome composition in our cohort was dominated by *Bifidobacterium* (mean abundance, standard deviation: 0.49 ± 0.33), followed by *Ruminococcus* (0.089 ± 0.140), Enterobacteriaceae (0.076 ± 0.085), and *Blautia* (0.037 ± 0.095) (**Figure 2A**). *Bifidobacterium*, the most abundant genus, was significantly enriched in OOM infants compared to ROC infants (p=0.03; Wilcoxon Rank-Sum) and elevated in non-allergic infants compared to those with atopic dermatitis and/or food allergy, although not significantly so (**Supplementary Figures 2A, B**). Microbiome alpha diversity did not differ significantly with respect to either population (p=0.15) or allergic outcome (p=0.20) in this sub-cohort (**Figure 2B**). Similarly, overall bacterial composition was not significantly different with respect to populations (p = 0.059; PERMANOVA) nor allergic outcome (p = 0.294) (**Figure 2C**).

### Microbiome IgA coating associates with populations and allergic outcomes

**Figure 3:**
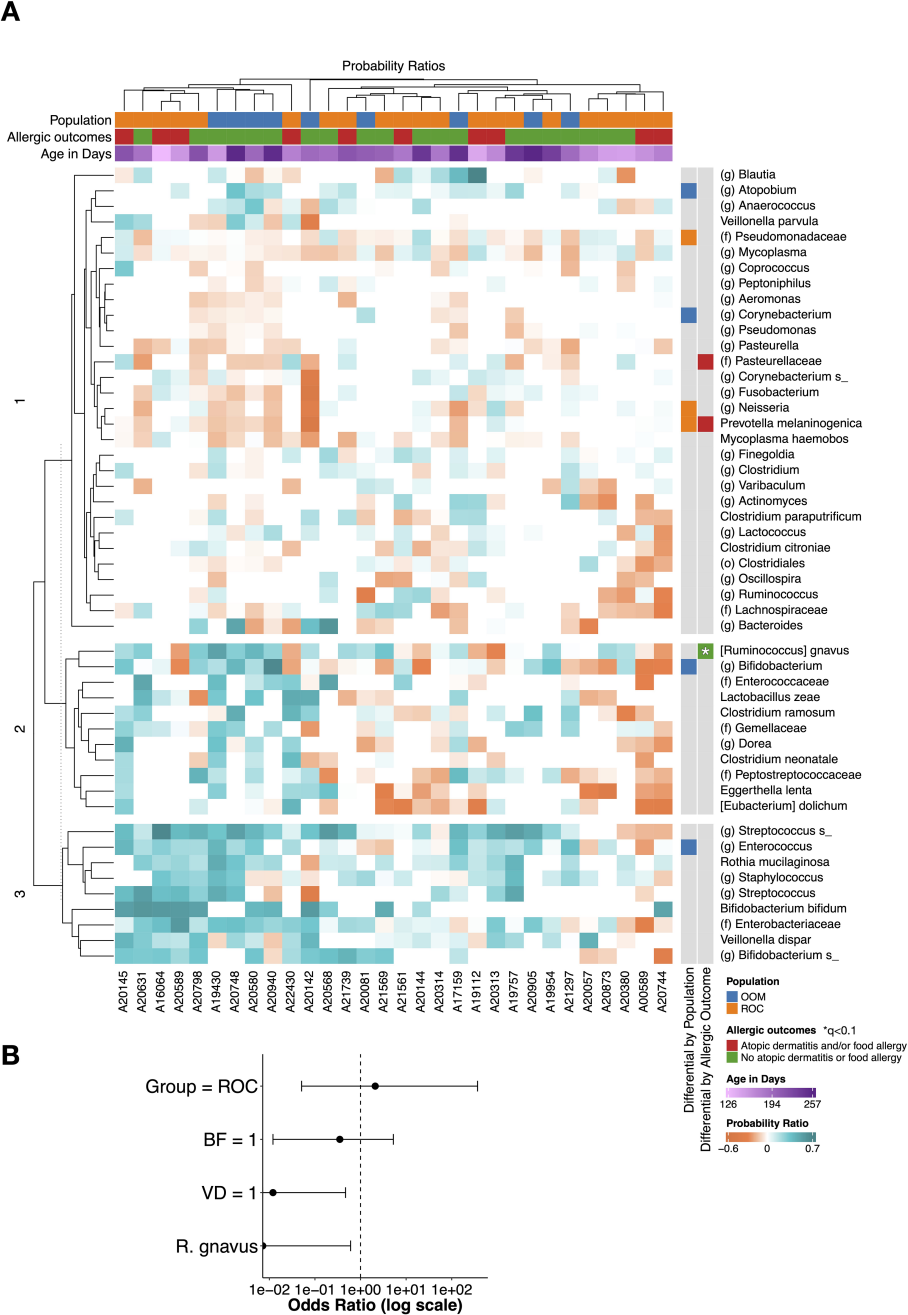
IgA coating of bacteria with respect to cohort, allergic outcomes, and demographic covariates. **(A)** Heatmap columns represent samples and rows represent taxa. Each cell depicts the IgA coating level of a given taxa in each sample. Higher coating levels (ProbRatio > 0) is indicated by teal, whereas orange represents lower coating (ProbRatio < 0). The three top column annotations represent, in order, (1) population to which the sample belongs (OOM: blue (n=9); ROC: orange (n=21)), (2) allergic outcome status (AD and/or FA: red (n=10); neither: green (n=20)), and (3) age in days. The two right hand row annotations (left to right) represent, for each taxon, whether its IgA coating level was differential with respect to (1) population (OOM: blue; ROC: orange) and (2) allergic outcome (AD and/or FA: red; neither: green). In row annotations, a star denotes q<0.1 after FDR correction. **(B)** Firth logistic regression conducted on allergic outcome status as a function of population, delivery mode, breastfeeding status, and IgA coating levels of *R. gnavus*.

Samples had a mean coverage of 40,391 ± 11,533 reads/sample, and a total of 908 ASVs were detected. *Streptococcus* was, on average, the taxa with the highest probability ratio to be IgA coated (mean and standard deviation: 0.251 ± 0.240), followed by *Bifidobacterium bifidum* (0.1921 ± 0.251), Enterobacteriaceae (0.1446 ± 0.195), and *Rothia mucilaginosa* (0.1388 ± 0.146). Differential analyses identified several bacteria with enrichment of IgA coating in each population. *Atopobium*, *Corynebacterium*, *Bifidobacterium*, and *Enterococcus* were significantly more IgA coated in OOM, while Pseudomonadaceae, *Neisseria*, and *Prevotella melaninogenica* had higher IgA coating in ROC (**Figure 3A**, second rightmost column next to the heatmap). In infants with allergic outcomes, Pasteurellaceae and *Prevotella melaninogenica* had significantly higher IgA coating levels, while *Ruminococcus gnavus* had higher coating in infants with non-allergic outcomes (**Figure 3A**, rightmost column). This association between higher IgA coating of *R. gnavus* and non-allergic outcomes remained significant after FDR correction. To account for lifestyle covariates, we additionally constructed a logistic regression model to study the relation between allergic outcomes and population, delivery mode, breastfeeding, and IgA coating of *R. gnavus*. In this model, increased IgA coating of *R. gnavus* remained significantly associated with protection against allergies (OR<0.01, p=0.034), with vaginal delivery being also protective (OR = 0.012, p=0.011) (**Figure 3B**). Breastfeeding was protective and belonging to the ROC population was deleterious, although neither significantly so (OR = 0.351, p=0.447 and OR = 2.099, p=0.692, respectively) (**Supplementary Table 3**).

### Gut commensal bacterial IgA coating potentially associates with infant age

**Figure 4:**
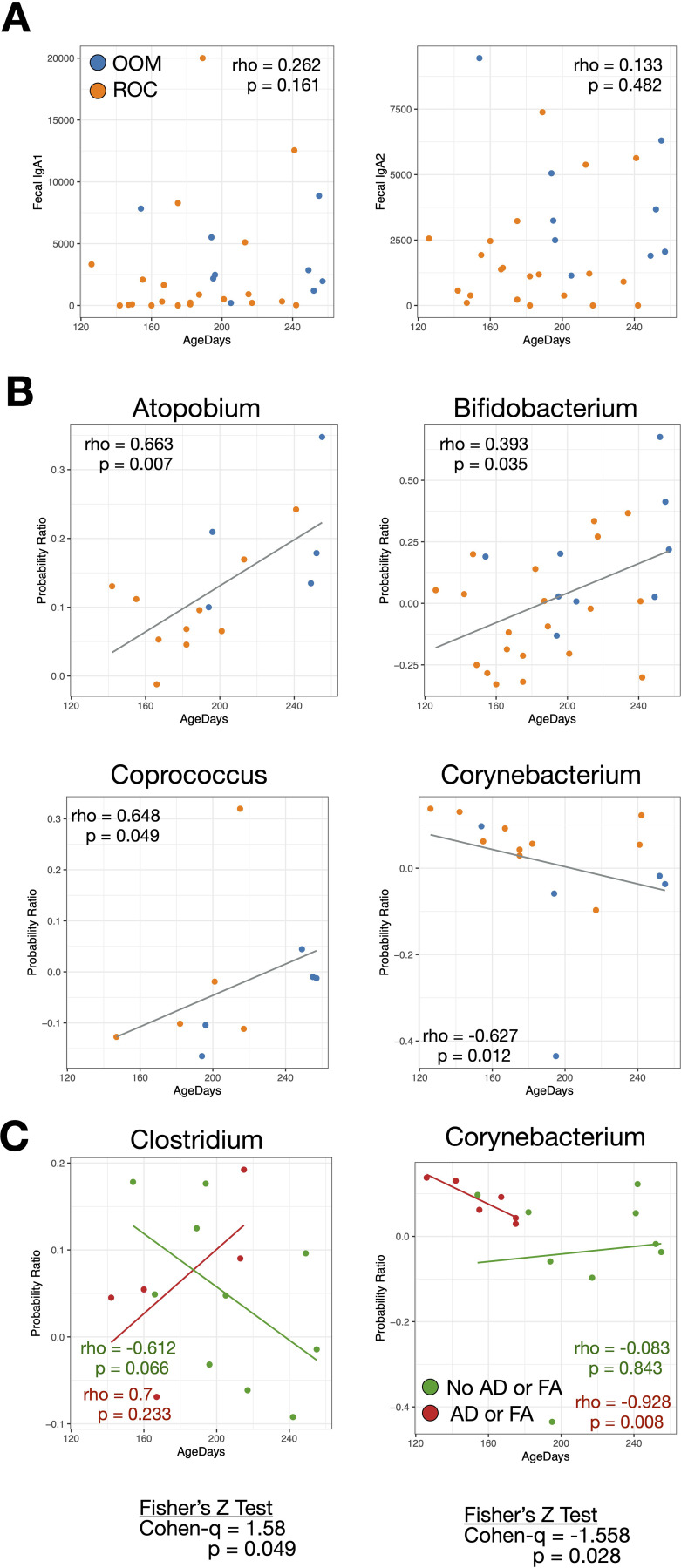
IgA coating as a function of age. **(A)** Fecal IgA_1_ and IgA_2_ in relation to infant age. **(B)** Bacterial taxa in which IgA coating is significantly associated with age. **(C)** Bacterial taxa in which IgA coating is differentially correlated with age with respect to allergic outcomes (atopic dermatitis and/or food allergy by 24 months).

The range of infant ages at the time of sample collection (120 to 260 days) allowed us to explore potential associations between fecal IgA levels, bacterial IgA coating, and infant age. While fecal IgA_1_ and fecal IgA_2_ were not significantly correlated with age (**Figure 4A**), several bacteria had significant changes in IgA coating over time. *Atopobium*, *Bifidobacterium*, and *Coprococcus* all had increased IgA coating with age, while *Corynebacterium* exhibited lower IgA coating in older infants (**Figure 4B**).

In addition, we found two bacteria in which the correlations between IgA coating and age were differentially associated with allergic outcomes. *Clostridium* had a decrease in IgA coating over time in non-allergic infants, but an increase in IgA coating in those with allergic outcomes (p=0.049, Fisher’s Z Test), while *Corynebacterium* had no change in IgA coating over time in infants with no allergic outcomes, and a decrease in coating in those with allergic outcomes (p=0.028) (**Figure 4C**).

### Human milk IgA binding to infant gut commensals associates with cohort and allergic outcomes

**Figure 5:**
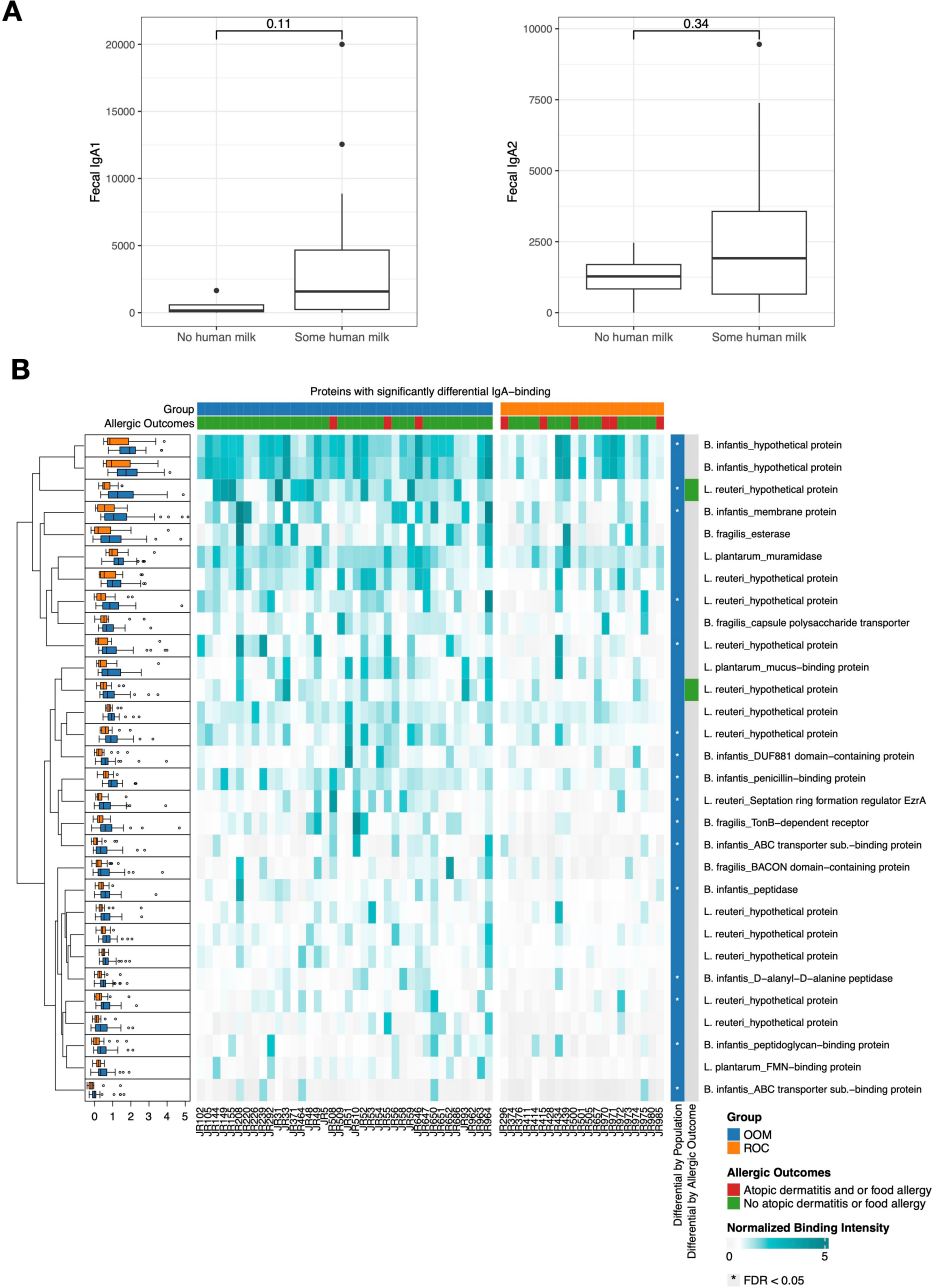
Fecal IgA levels with respect to dietary breastmilk and human milk IgA reactivity towards common infant gut commensals. **(A)** Fecal IgA_1_ and IgA_2_ with respect to breastfeeding. **(B)** IgA reactivity against antigen targets from *L. reuteri*, *B. infantis, B. fragilis* and *L. plantarum* in OOM (n=38) and ROC (n=21) milk samples with each column corresponding to a different sample. Column annotations denote group (OOM in blue, ROC in orange) and allergic outcome status (non-allergic in green, allergic in red) of the infant of the mother from which the human milk samples were derived. Rows depict antigens with boxplots in the left margin depicting distribution of data for each antigen with respect to cohort. Row annotations (right margin) denote row-wise direction of enrichment for each antigen; the second rightmost row annotation shows enrichment with respect to cohort (blue: OOM) with asterisks denoting significant after FDR correction (q<0.05). The rightmost row annotation shows enrichment with respect to allergic outcome.

In our cohort, we observed higher fecal IgA_1_ and IgA_2_ levels in breastfed versus non-breastfed infants, although the differences were not significant (p=0.11 and p=0.34 respectively; **Figure 5A**). We next explored the role of human milk IgA by measuring its binding capacity to antigens from specific infant gut bacteria. For this assay, we included *Bifidobacterium longum* subspecies *infantis* (hereafter, *B. infantis*), *Limosilactobacillus reuteri, Lactiplantibacillus plantarum*, and *Bacteroides fragilis* given their importance in early life, their potential immunomodulatory properties, and their relevance in the OOM population (see **Supplementary Materials**). Using an *in vitro* expression system coupled with a high−density microarray, we screened IgA binding to 454 proteins encoded by these bacteria. We used human milk samples collected at a median of 57 (ranging from 27-114) days of lactation across both populations (OOM n=38, ROC n=21). Out of 454 antigens tested, 87 were considered IgA-binding (see **Supplementary Materials**); of these, 30 exhibited differential IgA-binding capacity between OOM- and ROC-derived milk (p<0.05; Wilcoxon Rank-Sum). Among these 30 differential antigens, 16 remained significant after FDR correction (q<0.05) (**Figure 5B**, “Differential by population” column annotation). Milk from OOM mothers exhibited markedly higher IgA responses to antigens derived from these bacteria, with *B. infantis* and *L. reuteri* having the most antigens differentially recognized by milk IgA (13 and 10, or 43% and 33%, respectively).

We also assessed the IgA-binding capacity of human milk with respect to allergic outcomes in the matched infants. Among the 30 differential antigens, two proteins exhibited increased IgA binding to milk derived from mothers of non-allergic infants (p<0.05) (**Figure 5B**, “Differential by allergic outcome” column annotation). Both were hypothetical uncharacterized proteins from *L. reuteri* and their differences were not significant after FDR correction (q<0.05).

### *Bifidobacterium infantis* binds immunoglobulins in an fab-independent manner

To measure IgA binding intensity to whole bacteria, we next grew *L. reuteri* and *L. plantarum* cultures for 48 hours, incubated them with human milk samples, stained for IgA binding, and performed flow cytometry-based assessment of IgA binding. We found no significant differences in levels of IgA binding between OOM and ROC milk samples for either *L. plantarum* or *L. reuteri* (p=0.8737 and p = 0.7979, respectively), although *L. reuteri* had significantly higher IgA binding than *L. plantarum* (p<0.005) (**Supplementary Figure 3**).

We further investigated IgA binding of *B. infantis* whole bacteria grown for 48 hours. We noted that *B. infantis* seemed to grow in two distinct populations, based on the nucleic acid stain SYTO-BC (we hereafter denote these two sub-populations as SYTO-BC^hi^ and SYTO-BC^lo^). Interestingly, in the absence of human milk, *B. infantis* consistently generated a strong fluorescent signal upon staining with our Alexa-conjugated anti-IgA detection antibody. To evaluate whether the observed effect was attributable to inadequate blocking, we replaced the standard blocking reagent with 2% unlabeled goat IgA, applied as a cold block before incubation with the fluorescent goat anti-human IgA antibody (goat IgG isotype). This modification did not alter the outcome, indicating that the finding was not due to improper blocking. Additionally, since *B. infantis* has been previously described to have the ability to utilize N-glycans (35), which are also present in the structure of immunoglobulins, we sought to remove glycan motifs from the PE-conjugated IgA to test whether this modification abrogated binding. We enzymatically deglycosylated the Fc region of the anti-IgA detection antibody, confirmed the removal of N-glycans by observing the characteristic downward shift on SDS–PAGE and then repeated the staining procedure under both standard serum and 2% IgA block conditions. Because our initial assays used formalin-fixed bacteria to halt growth and ensure biosafety, we also considered that fixation might expose or alter bacterial surface epitopes, enhancing nonspecific interactions. We thus compared eight experimental conditions to combinatorially assess the effects of blocking, fixation, and deglycosylation on immunoglobulin binding. We found that for the SYTO-BC^lo^ population, deglycosylation significantly reduced the IgA-binding capacity of *B. infantis*, while buffer composition and fixation did not significantly affect the binding (**Supplementary Figure 4A**). SYTO-BC^hi^ IgA binding was negligible for this analysis.

Finally, we sought to understand whether the growth phase of *B. infantis* was related to the differentially binding populations. We tested the IgA-binding ability of the SYTO-BC^lo^ and SYTO-BC^hi^ populations over the 71h course of growth phase of *B. infantis*. We found that the IgA binding of the SYTO-BC^lo^ population increased consistently over time, while the SYTO-BC^hi^ population increased negligibly (**Supplementary Figure 4B**). Assuming no time-dependence in the IgA-binding affinity of both populations, this suggests that the SYTO-BC^lo^ population grew at a slower rate than the SYTO-BC^hi^ population and that the SYTO-BC^hi^ population exhibited reduced IgA-binding ability compared to the SYTO-BC^lo^ population.

## Discussion

To our knowledge, our study is the first to assess the role of bacterial IgA coating in allergic outcomes at 2 years of age in a birth cohort that includes Old Order Mennonites, a population with a traditional farming lifestyle and significantly lower prevalence of allergies (16). We identified bacteria with differential IgA coating levels associated with both population of origin and allergic outcome. Specifically, we found *Atopobium*, *Corynebacterium*, *Bifidobacterium*, and *Enterococcus* had higher coating in OOM infants, while Pseudomonadaceae, *Neisseria*, and *Prevotella melaninogenica* had higher coating in Rochester infants. *Atopobium* has been previously found to be negatively correlated with both serum mite-specific and fecal IgE levels (36) while *Bifidobacterium* has been associated with protection against atopic diseases (10, 37), and infants with higher levels of *Corynebacterium* exhibit reduced asthma severity (38). We additionally found *P. melaninogenica* was highly coated in infants with allergic outcomes, while *Ruminococcus gnavus* had higher coating in those without allergies. Interestingly, infants with cow milk allergy and atopic dermatitis in Qatar also exhibited higher IgA coating of *P. melaninogenica* (39). After adjusting for lifestyle factors in our cohort, the potential protective effect mediated by *R. gnavus* remained significant using a logistic regression model controlling for population, delivery mode, and breastfeeding. Vaginal delivery was also significantly protective, with breastfeeding and belonging to the OOM population also having a protective effect although not statistically significant. While previous work has identified some of these factors as important in allergic outcomes (40), the moderate sample size of our cohort and the lack of external validation suggest these associations are hypothesis-generating and will require further study.

Previous studies in infants that developed asthma by 7 years of age found reduced coating of *Faecalibacterium* and *Bacteroides* and higher coating of Lachnospiraceae associated with allergic manifestations (eczema, allergic sensitization, asthma, allergic rhinitis, allergic urticaria), as well as higher coating of Erysipelotrichaceae in healthy children (41). This study, however, was part of an intervention to test the potential protective effect of *Limosilactobacillus reuteri* ATCC 55730 in at-risk infants with a familial history of allergic disease, representing a study design different from ours. A study of celiac disease in Swedish infants found highly coated *Actinomyces*, *Coprococcus comes*, Ruminococcaceae, and *Prevotella copri* at 2.5 years of age in those who progressed to develop the disease (42), highlighting some commonalities with our own findings although in a different condition and age range. In a cohort of Peruvian adolescents aged 9 to 17, asthma was associated with reduced coating of *Blautia*, *Ruminococcus*, and Lachnospiraceae (43). Importantly, these prior studies targeted populations with demographic differences to ours (older subjects and distinct ethnicities) and utilized IgA coating metrics that have specific biases (44), while we employ a more recent approach that has been shown to be methodologically robust (45).

We also identified specific microbes in which IgA coating levels were correlated with infant age. IgA coating levels of *Atopobium*, *Bifidobacterium*, and *Coprococcus* increased with infant age, while IgA coating levels of *Corynebacterium* decreased with increasing age. However, due to the moderate sample size of our cohort and the fact that these correlations were derived from cross-sectional samples from different individuals rather than longitudinal samples of the same infants, we acknowledge that these associations would require further validation. For instance, findings for *Coprococcus* are based on only ten data points, while the correlations involving *Atopobium, Bifidobacterium*, and *Corynebacterium* were estimated from 15 or more data points. Furthermore, samples in our study were obtained within a limited range of ages, spanning only days 120–260 in early infancy.

In previous work characterizing IgA coating within the first year of life, a study of twin pairs recruited from the general population found an increase in IgA coating of *Bifidobacterium longum* within the first year of life (46). Another study of infants sampled longitudinally across different countries and in the context of diabetes observed increases in IgA coating of members of the Bifidobacteriaceae and Lachnospiraceae families (to which *Coprococcus* belongs) within the 100–200 day time window, comparable to the time frame of samples in our study (47). These findings combined with our results suggest that microbiome maturation can be partially driven by immune responses to colonization, which might have important consequences for allergic disease risk (48). Previous efforts at creating a microbiome “maturation index” emphasized the importance of *Bifidobacterium* and Bacteroides as critical taxa in establishing early microbial communities (45). We hypothesize that IgA coating of healthy commensals such as *Bifidobacterium* promotes immune tolerance and enables these keystone species to persist and shape the infant microbiota.

Given that prior literature has suggested a protective role for *Bifidobacterium* (10, 37) as well as *Lactobacilli* (currently named *Limosilactobacilli* and *Lactiplantibacilli*) in allergic outcomes (49, 50), we supplemented findings from our high-throughput IgA-SEQ analyses with *in vitro* characterization of the IgA-binding capacity of several strains belonging to these taxa. Selection of these strains was informed by prior studies that demonstrated potential immune benefit of these taxa in promoting positive health outcomes and healthy gut microbiome colonization in infants, such as the SEPSiS trial (51). Because human milk represents a major source of intestinal IgA in early infancy, we used a protein microarray to characterize IgA-binding capacity of paired maternal milk in the OOM and ROC populations. We found that human milk IgA from OOM mothers binds significantly more to proteins from *L. reuteri*, *L. plantarum*, *B. fragilis*, and *B. infantis* compared to milk IgA from ROC mothers. However, when we utilized flow cytometric assays to measure the IgA binding towards cultured whole strains, these differences were not observed for *L. reuteri* or *L. plantarum.* It is possible that the proteins utilized for the protein array were not expressed on the cultured whole bacteria, resulting in the reduced binding capacity observed, or that specific characteristics of strains utilized across the assays were responsible for these differences. We did note, however, that ATCC *L. reuteri* binds to human milk IgA significantly more than ATCC *L. plantarum*. This suggests that intrinsic bacterial surface factors, such as extracellular binding proteins or exopolysaccharides (49, 52), may be driving IgA recognition more than variations in milk composition or IgA concentration.

A highly unexpected finding was that *B. infantis* directly binds to our anti-IgA detection antibody (goat polyclonal anti-human IgA) in the absence of milk IgA, which was not observed with *L. reuteri* or *L. plantarum*. Deglycosylation of the detection antibody partially attenuated binding, suggesting a glycan−dependent mode of Ig engagement by *B. infantis*. This interaction likely involves membrane surface proteins on the bacterium recognizing N−glycans. Previous work has shown that *B. infantis* harbors an expanded repertoire of glycan- and solute-binding proteins and lectin-like adhesins that recognize host N-glycan structures (53). Our findings support a non-classical mechanism whereby *B. infantis* binds IgA via immunoglobulin-associated glycans, explaining why removing those glycans reduces bacterial attachment. Increased immunoglobulin binding to these strains may thus help facilitate their colonization while also potentially providing an additional carbon source. It must be noted, however, that while binding was significantly reduced, it was not fully eliminated, suggesting that additional non-glycan–mediated interactions may play a role in immunoglobulin binding to *B. infantis*. Given that the binding is Fab-independent, it may be appropriate to assess binding in these experiments as total immunoglobulin binding as opposed to specifically IgA binding. This observation is consistent with previous findings, as it has been shown that the interaction between commensals and secretory IgA (SIgA) is not only antigen-driven (54), as natural polyreactive SIgA and Fab/Fc-independent, glycan-mediated binding also likely contribute substantially to this process (55). Deciphering these mechanistic interactions between the IgA and commensal bacteria is critical for understanding downstream effects such as epithelial cell programming, maintenance of tissue homeostasis, and the regulation of immune maturation (56, 57).

Our flow cytometry analysis of *B. infantis* ATCC 15697 uncovered two distinct populations—SYTO-BC^hi^ and SYTO-BC^lo^—with the latter exhibiting substantially greater IgA binding at all sampled time points. The SYTO-BC^lo^ cells may represent a physiological state characterized by reduced DNA staining and upregulated lectin-like surface proteins. While flow cytometry revealed distinct SYTO-BC^hi^ and SYTO-BC^lo^ subpopulations, we have not yet performed fluorescence-activated cell sorting (FACS) to isolate and characterize these different populations. These include high-resolution imaging (e.g., confocal microscopy) to examine cell-surface architecture that correlates with antibody binding or pili-specific staining to discern their role in the N-glycan-mediated immunoglobulin binding we observed in these strains. Lastly, given the Fab-independent binding seen by *B. infantis*, utilization of a human milk-purified IgA directly conjugated to a fluorophore may assist in further analysis of the binding capacity of these strains.

Limitations of our work include small sample size and the assessment of microbial composition with IgA-SEQ, which combines flow cytometry with 16S rRNA gene sequencing and limits our ability to distinguish bacterial strains. The cross-sectional design of our study and the lack of external validation prevent us from determining the extent to which the observed association between IgA coating of specific bacteria and infant age are generalizable.

Overall, findings from our study point to the role of IgA coating of bacterial taxa in identifying microbes with potential immunomodulatory roles in a population of low allergic risk. We further identify microbes in which IgA coating is potentially associated with age, suggesting a complex relation between lifestyle and environmental factors, IgA production, and shaping of microbial communities over the first year of life. Future studies incorporating longitudinal sampling of infants will be required to confirm our findings and their relation to allergic outcomes.

## Methods

### Study populations and sample collection

Samples used for IgA-SEQ characterization are derived from a longitudinal birth cohort established in Western New York called “Zooming into Old Order Mennonites” (ZOOM), as described in prior work (34). We selected a total of 30 infants who had provided stool samples, from the Old Order Mennonite (N = 9: no allergies at 24 months) and from the Rochester population (N = 10: allergies at 24m; N = 11 no allergies at 24m).

### DNA extraction and sequencing

DNA extraction and sequencing library preparation was performed as previously described (7). Briefly, stool samples were suspended and centrifuged; the pellet was reconstituted with the addition of lysis buffer and homogenized via bead beating. The aqueous phase was washed in a phase separation tube and DNA was separated by alcohol precipitation. Sequencing was performed on the Illumina MiSeq instrument with 2x250bp reads, targeting the V4 region of the 16S rRNA gene following previously published protocols (58).

### IgA-SEQ analysis

Microbiome analyses were performed using Qiime2 v2020.8.0 and taxonomic assignment was done using GreenGenes1 (59, 60). IgA coating was quantified using the IgA Scores v0.1.2 R package and the Probability Ratio metric (44).

### Human milk IgA binding to infant bacterial antigens

Reactivity of human IgA was assessed as previously described (61). Briefly, genome libraries were constructed by cloning selected open reading frames (ORFs) from selected commensal organisms based on prior studies (23, 51, 62, 63). Proteins encoded by these ORFs were expressed using an *E. coli in vitro* transcription/translation system. Microarrays were incubated with antibody-containing samples and bound antibodies were detected using a fluorescent detection antibody towards IgA.

### Flow-based IgA binding of infant gut commensals

Flow cytometry was performed to assess the binding of IgA towards *L. reuteri* (ATCC 23272), *L. plantarum* (ATCC 202195), and *B. infantis* (ATCC 15697) utilizing human milk as a source of IgA.

### Statistical analyses

Association between categorical variables was tested using Fisher’s Exact Test. Comparisons of continuous variables between groups were done using the Wilcoxon Rank-Sum or T-Test when appropriate. Firth Logistic Regression was used to model allergic outcome as a function of covariates and IgA coating of taxa using the formula: AllergicOutcome ~ SomeBF + DeliveryMode + Group + ProbRatio(*R.gnavus*).

A full description of the cohort, methods used for sample collection, DNA extraction and sequencing, as well as the antigen-binding assay and all statistical analyses are provided in **Supplementary Materials**. Sequencing data has been uploaded to PRJNA1320699.

## Data Availability

The datasets presented in this study can be found in online repositories. The names of the repository/repositories and accession number(s) can be found below: https://www.ncbi.nlm.nih.gov/, PRJNA1320699.
