# Supplementary material for: Gut microbial IgA coating in infants with traditional farming lifestyle and urban infants with allergic outcomes
Source: Front Immunol. 2026 Apr 20;17:1793302. doi: 10.3389/fimmu.2026.1793302 (PMC13136260; doi:10.3389/fimmu.2026.1793302)
Supplement: Supplementary file 1 [file DataSheet1.pdf]

SUPPLEMENTAL MATERIALS

Study populations and sample collection. Samples used for IgA-SEQ characterization are derived from a longitudinal birth cohort established in Western New York called "Zooming into Old Order Mennonites" (ZOOM), as described in prior work [1]. This cohort was recruited prenatally from two communities with a different lifestyle due to their differing risk for allergic diseases: infants born to OOM mothers from Yates County and surroundings who are at low risk for allergic diseases (N=76) and those with a first-degree biologic family member with history of allergic disease born to ROC urban/suburban mothers (N=70). Subjects were followed for 24 months for clinical data, presentation of allergic outcomes and collection of various biological samples at birth, 1-2 and 6 weeks, and 6, 12, 18, and 24 months. Allergic outcomes including atopic dermatitis, food allergy, allergic rhinitis and wheeze were diagnosed by a physician throughout the first 24 months of life based on clinical presentation and skin prick testing and/or specific IgE and oral food challenges as clinically indicated. Specific IgE to major food and aeroallergens was performed on all to assess sensitization. Allergic outcomes most common in the first 24 months of life were atopic dermatitis and food allergy, and a focus of the current analyses. For the purposes of IgA-SEQ analyses presented here, we down-selected a total of 30 infants to have a smaller but representative cohort of stool samples from OOM (N=9), and from both ROC infants who developed (N=10) or did not develop (N=11) allergic diseases. Of the total of 10 infants who developed allergic outcomes, all had atopic dermatitis and five also had food allergy. Stool samples were collected from infants as previously described, then immediately frozen in home freezers and transported to University of Rochester Medical Center for long term storage in freezers at -80C until processed for microbiome characterization.

Genomic DNA extraction and sequencing from fecal samples. ~100 mg of stool was suspended in 1 mL of PBS and pelleted by centrifugation at 10000g for 10 min, 4°C. Supernatant was removed, the bacterial pellet reconstituted in 400 μL of PBS, transferred to 2 mL Lysing Matrix B tubes (MP Biomedicals, Eschwege Germany) and 300 μL of lysis buffer (680 mM sodium chloride, 680 mM Tris-HCl, 68 mM EDTA, pH 8.0), 200 μL 20% SDS and 500 μL of phenol-chloroform-isoamyl alcohol (25:24:1) was added. Samples were subjected to homogenization by bead beating on a Bead Mill (VWR, Radnor PA) (3 cycles of 30s with 4 m/s), aqueous phase was separated and washed with 750 μL of phenol-chloroform-isoamyl alcohol in a phase separation tube (5PRIME, low density tube). DNA was then separated by alcohol precipitation. The V4 region of the 16S rRNA DNA gene was amplified in triplicate from all samples, pooled, and sequenced with the Illumina MiSeq platform (paired-end, 250bp) following standard protocols as previously described [2; 3]. The primers used were Cdf 5'-AATTGACGGGGGCCCGCACAAG-3' and Fba 5'-TGACGACAACCATGCACC-3'.

IgA-Seq:

Sample preparation and staining: Single cell suspension was prepared from stool samples by resuspending 50mg of previously frozen stool into 0.5 mL cold PBS in a Lysing Matrix D tube (MP Biomedicals). The tube was incubated on ice for 60min, homogenized in Bead Mill (3 cycles of 30s at 4 m/s) and centrifuged 50g 15 min at 4 °C. The supernatant, containing bacterial single cell suspension was separated, an aliquot stored for further analysis, and the cells were pelleted by centrifugation at 8000g, 5 min at 4 °C and the supernatant was removed. Cells were then washed twice with staining buffer (1% BSA in PBS) by resuspending and pelleting as above. Finally, cells were resuspended in 90 µL of staining buffer and 10 µL of PE-anti human IgA (Miltenyi Biotech), incubated for 30 min on ice, and washed three times with 1 mL of staining buffer as above. Cells were then resuspended in 1 mL of staining buffer and 50 µL of anti-PE microbeads (Miltenyi Biotech) and incubated on ice for 15 min. Labeled cells were then washed twice with 1 mL cold staining buffer as above.

MACS separation: For IgA coating-based purification, labeled cell suspension was passed through a Miltenyi LS column, previously equilibrated with staining buffer and placed in a Miltenyi magnetic holder. Column was washed with 2 mL of staining buffer and flowthrough and washes were combined as IgA negative fraction. Column was then removed from the magnetic holder, and bound, IgA positive bacteria were eluted using 3 mL of staining buffer and the plunger provided with the column. Cell suspensions were aliquoted for further analysis and immediately run on analytical flow cytometry or stored frozen for genomic DNA extraction and bacterial composition analysis.

Analytical FACS: For sample purity verification by analytical flow cytometry, aliquots of cell suspensions were stained for presence of DNA by using SytoBC (Invitrogen). This was done by resuspending pelleted sample into 1:1000 dilution of SytoBC stain in PBS, followed by incubation for 30 min at 4°C. Samples were then washed in PBS once and re-stained by PE-anti-IgA as above. Flow cytometry was performed using Becton-Dickinson LSR flow cytometer. See **Supplementary Figure 2** for example of flow results.

Taxonomic, IgA-SEQ, and Statistical Analyses. Initial microbiome analyses were performed using QIIME2 v2020.8.0 with DADA2 and default parameters, unless noted otherwise [4]. Taxonomic assignment was performed using GreenGenes1 [5]. ASV tables were rarefied at 27,149 sequences per sample before calculating diversity metrics. This rarefaction depth was chosen based on the minimum sequencing depth that would retain all 30 samples. Alpha diversity was estimated using Faith's phylogenetic diversity [6], while beta diversity was estimated with unweighted UniFrac [7]. Alpha rarefaction curves for Faith's PD showed a stable diversity estimate for

samples above 10,000 reads confirming 27,149 as an appropriate rarefaction depth. Differences in alpha and beta diversity between groups were tested using the ANOVA and PERMANOVA tests with 1000 iterations. IgA coating was quantified with the *IgA Scores* R package v0.1.2 with the Probability Ratio (ProbRatio) metric [8]. If a taxon was not detected in either the IgA-positive or IgA-negative fraction, it was assigned a ProbRatio value of NA. Differences in IgA coating between groups were tested using the Wilcoxon Rank-Sum test. P-values were adjusted using FDR (BH) and a multiple comparisons threshold of $q<0.1$ was used. Correlations were estimated using the Spearman correlation coefficient, and differential correlations were tested using the R package *diffcor* v0.8.2. Firth logistic regression was conducted using the R package logisf 1.26.0 [9; 10]. Heatmaps were generated with ComplexHeatmap v2.20.0, and other plots were generated using ggplot2 v3.5.1. Taxa represented in the heatmap were obtained by filtering features with near zero variance using the package nearZeroVar 6.0 with freqCut=95/5 (default) and uniqueCut=30. All R analyses were performed in R v4.4.3.

Protein array assessing human milk Ig binding to infant gut commensals.
Reactivity of human IgA was assessed in collaboration with Antigen Discovery, Inc, as described before [11]. Antigen targets in this study include 454 proteins from common infant commensals including *L. reuteri*, *B. infantis, B. fragilis* and *L. plantarum.* Commensal bacteria were selected based on taxa that were most explored in previous trials assessing infant immune health and health benefits. For example, *Bifidobacterium* was shown to have potential immunomodulatory benefit in infants [12]. *L. reuteri* was shown to be more highly coated in OOM mothers compared to ROC mothers [13]. *L. plantarum* (ATCC 202195) was shown in the Synbiotics for the Early Prevention of Severe Infections in Infants (SEPSiS) trial to have benefit in newborns by promoting healthy gut colonization and reducing infection [14]. Finally, *B. fragilis* has been shown to utilize IgA coating for gut colonization and was thus included in the array [15]. Genome libraries were constructed by cloning selected open reading frames (ORFs) from selected commensal organisms. The clone library was created through an *in vivo* recombination cloning process with PCR-amplified coding sequences, and a complementary linearized expressed vector transformed into chemically competent *E. coli* cells was amplified by PCR and cloned into the pXI vector using high-throughput PCR recombination cloning. Proteins encoded by these ORFs were expressed using an *E. coli in vitro* transcription/translation (IVTT) system, and individual proteins were printed onto individual nitrocellulose-coated glass AVID slides (Grace Bio-Labs, Inc., Bend, OR) using an OmniGrid accent robotic microarray printer (Digilabs, Inc., Marlborough, MA). Microarrays were incubated with a 1:25 dilution of antibody-containing samples (milk or serum) that were pre-incubated with a DH5α *E. coli* lysate solution at 3mg/mL, washed, and bound antibodies were detected using an α chain-specific Cy3-conjugated fluorescent detection antibody towards IgA (Jackson

ImmunoResearch, West Grove, PA, Cat#109-166-011). Microarray slides were scanned on a GenePix 4300A high-resolution microarray scanner (Molecular Devices, Sunnyvale, CA), and the scanned images were quantified using Mapix software (Innopsys, Carbonne, France). All further data processing was performed in R. Data were normalized by first transforming raw values using the base 2 logarithm. Next, the data set was normalized to remove systematic effects by subtracting the median signal intensity of the IVTT control spots for each sample. This procedure normalizes the data and provides a relative measure of the specific antibody binding versus the nonspecific antibody binding to the IVTT controls. With the normalized data, a value of 0.0 means that the intensity is no different than that of the IVTT controls, and a value of 1.0 indicates a doubling with respect to IVTT control spots. Differential IgA reactivity between OOM and ROC cohorts was assessed by the Wilcoxon Rank-Sum Test, and P-values were adjusted for the false discovery rate using the method described by Benjamini and Hochberg [16]. Heatmaps were generated with ComplexHeatmap v2.20.0 [17].

Flow-based IgA binding of infant gut commensals. Flow cytometry was performed to assess the binding of IgA towards *L. reuteri* (ATCC 23272), *L. plantarum* (ATCC 202195), and *B. infantis* (ATCC 15697) utilizing human milk as a source of IgA. OD_{600} ~ 0.5 bacterial cultures were diluted 1:100 in 500 µL of MRS Broth (*L. reuteri* and *L. plantarum*) or RC Broth (*B. infantis*) and pelleted via centrifugation at 10,000g for 10 minutes at 4 °C. Supernatant was decanted. Pelleted cells were resuspended in either 500 µL of 1x Phosphate Buffered Saline (PBS, Sigma-Aldrich Co., St. Louis, MO) or 1X PBS supplemented with 55 µL of 10% formalin. Resuspended bacteria were incubated for 10 min at 4°C and then pelleted via centrifugation at 10,000g for 10 minutes at 4 °C. Samples were resuspended and blocked with blocking buffer (10% Goat Serum, two mM EDTA in 1x PBS) for one hr. at 4°C. Test samples were resuspended in 50 µL of milk (stocks stored at -80 °C), diluted 1:1 in 1x PBS, while milk-free controls were suspended in 50 µL of 1x PBS; tubes were incubated at 4 °C for 1 hr. Tubes were diluted with 950 µL of 1x PBS and pelleted via centrifugation at 10,000g for 10 min at 4°C and supernatant was decanted. Test samples were stained with 50 µL of SYTO-BC (ThermoFisher Scientific, Waltham, MA) diluted 1:100 in blocking buffer and negative control with 50 µL of 1x PBS; tubes were incubated for 30 min at 4°C. Samples were pelleted via centrifugation in at 10,000xg for 10 minutes at 4°C and stained with 50 µL staining antibodies diluted in staining buffer and incubated for 30 min at 4 °C: 1:250 dilution of PE-conjugated goat polyclonal IgA antibody (PE; Southern Biotech, Birmingham Alabama), 1:10 dilution of APC-conjugated mouse monoclonal lambda light chain antibody (APC; Life Technologies, Carlsbad, CA), 1:20 dilution of APC-conjugated mouse monoclonal kappa light chain antibody (APC; Life Technologies), and 1:50 dilution of brilliant violet 421 (BV421)-conjugated rat monoclonal IgG antibody (BV421; Biolegend, San Diego, CA). Tubes include All-stains, stains individually, and fluorescent minus one (FMO) control. Samples were centrifuged at 10,000g for 10 min at 21 °C,

decanted, resuspended in 500 µL 1x PBS, and transferred to flow cytometric tubes. Fluorescent intensity was measured using an LSR II Flow Cytometer (Becton Dickinson) and analyzed using FCS Express Flow Cytometry Software (De Novo Software; Glendale, CA). Final values were expressed as median fluorescent intensity (MFI) of populations gated for SYTO-BC^{+}/IgA+ or SYTO-BC^{+}/IgG^{+} populations. Statistical analysis (Student's T-test and One-Way ANOVA) was performed using GraphPad Prism.

SUPPLEMENTAL FIGURES

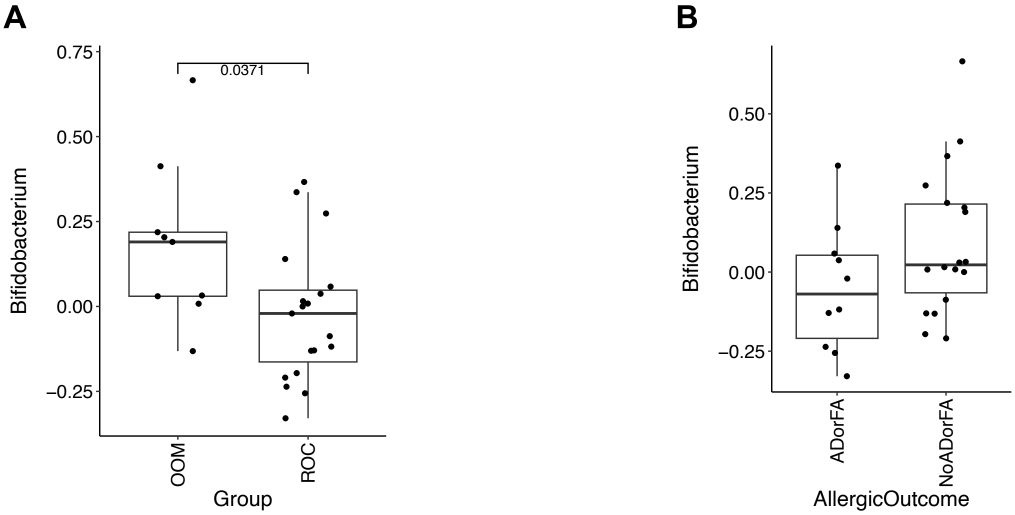

S1. *Bifidobacterium* relative abundances with respect to population and allergic outcome. Relative abundance with respect to (A) population, i.e. OOM vs ROC, (B) allergic status i.e. having atopic dermatitis and/or food allergy versus neither of the two.

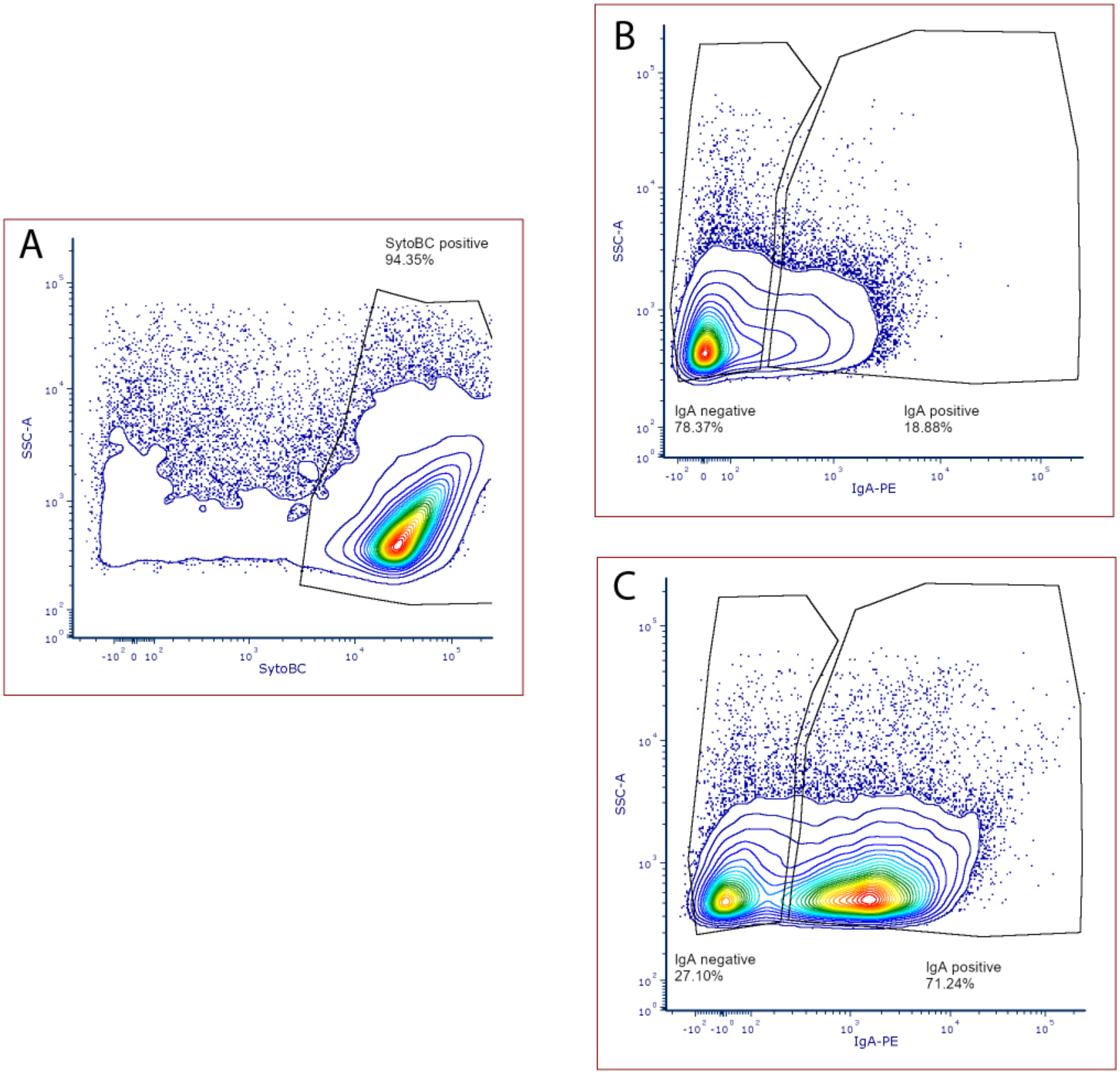

S2. FACS characterization of IgA sorted bacteria from stool samples. Analytical flow was performed to assess the quality of MACS sorted fecal bacteria. A representative example is shown. **A.** Syto BC staining was used to differentiate DNA containing bacteria from debris. IgA staining is then visualized for MACS based IgA negative (**B**) and IgA positive (**C**) fraction.

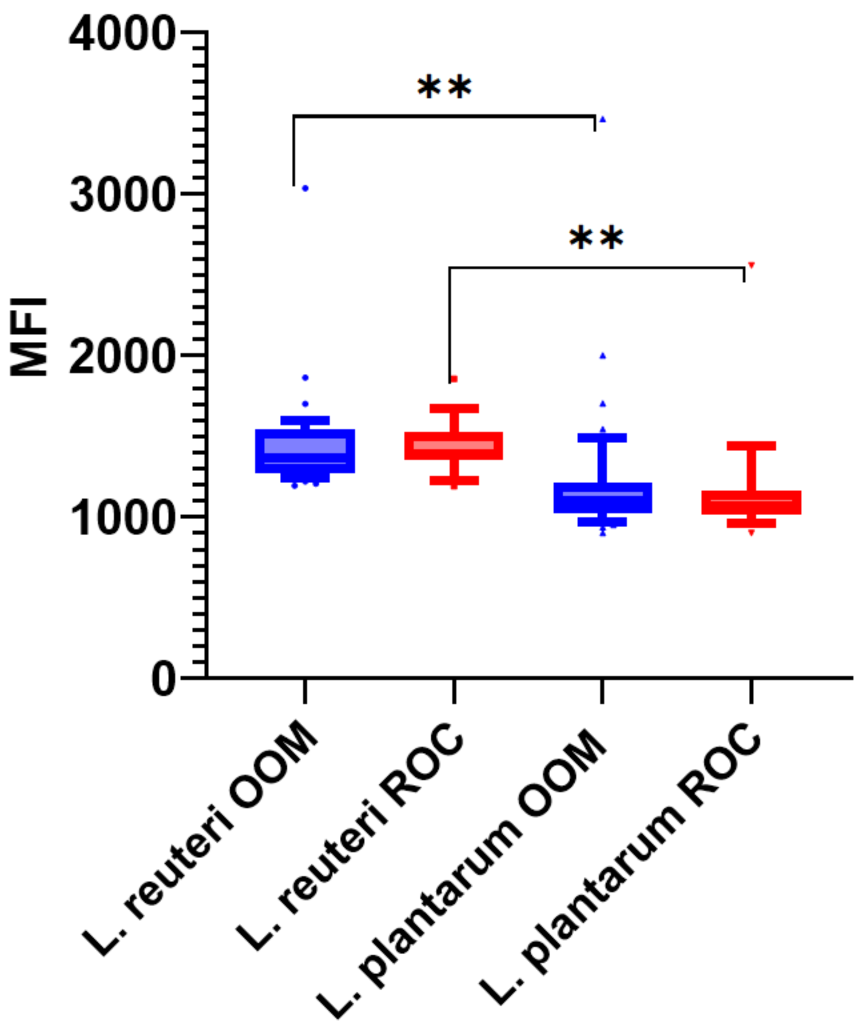

S3. Human milk IgA-binding intensity toward *L. plantarum* and *L. reuteri*. Box plot showing IgA-binding intensity of human milk IgA from OOM (n = 38) and ROC (n = 21) cohorts towards *L. reuteri* and *L. plantarum*. Intensity determined as a median fluorescent intensity (MFI) of the SYTO-BC+/IgA+ cell population gate.

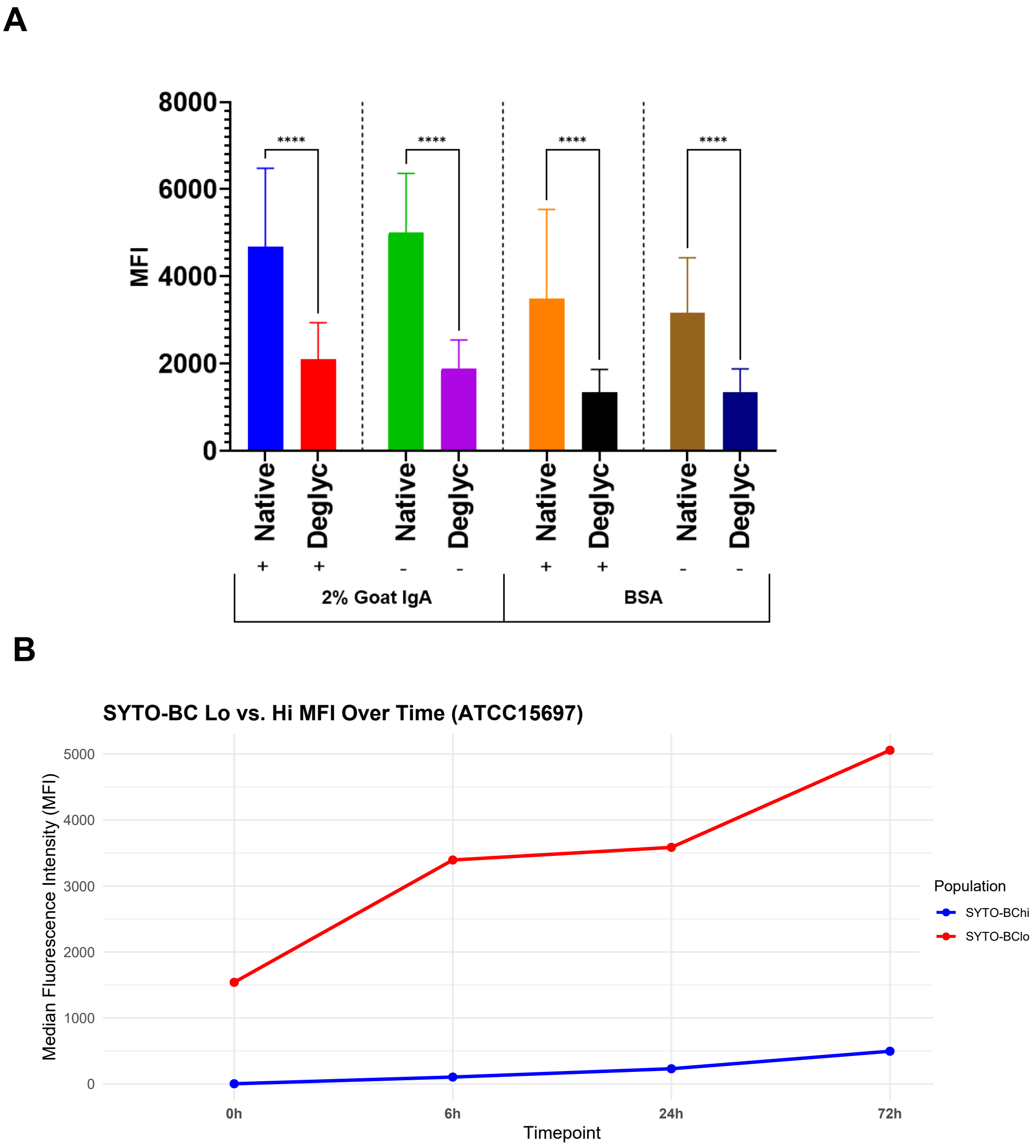

S4. *B. infantis* binds immunoglobulins in a Fab-independent manner. (A) *B. infantis* immunoglobulin A binding is abrogated by enzymatic deglycosylation. Plot showing the immunoglobulin binding capacity of *B. infantis* (SYTO-BC^{lo}), either native or deglycosylated PE-IgA antibody. The experiment was performed using BSA or 2% unlabeled goat IgA blocking reagents (labeled below corresponding groups of 4) on either fixed (+) or unfixed (-) cells. **(B)** *B. infantis* strain ATCC15697 has a secondary population with reduced immunoglobulin binding. *B. infantis* ATCC15697 was cultured for 72 h, with aliquots taken at 0, 6, 24, and 72 h for flow-cytometric staining. The plot shows median fluorescence intensity (MFI) of the two IgA-positive populations: SYTO-BC^{lo} (red) and SYTO-BC^{hi} (blue).

SUPPLEMENTARY TABLE 1

Covariates	ADorFA (N=11)		NoADorFA (N=19)		P-value
**Age (days, median ± s.d.)**	175 ± 28		196 ± 37		0.020
**Male / female**	4 / 7		4 / 15		0.417
**Vaginal / C-section**	6 / 5		19 / 0		0.15
	Yes	No	Yes	No	
**Antibiotics**	2	9	4	15	1.0
**Some breastfeeding**	8	3	18	1	0.126
**Cat in household**	2	9	11	8	0.057
**Dog in household**	8	3	12	7	0.702

Supplementary Table 1. Cohort demographics with respect to allergic outcomes for samples used for IgA-SEQ. Cell values indicate number of infants in each cohort corresponding to each covariate. P-values indicate tests of association using Student's T-Test for continuous variables and Fisher's Exact Test for categorical variables.

SUPPLEMENTARY TABLE 2

Covariates	ADorFA (N=11)		NoADorFA (N=10)		P-value
Age (days, median ± s.d.)	175 ± 28		196 ± 37		0.141
Male / female	8 / 3		9 / 1		0.586
Vaginal / C-section	6 / 5		10 / 0		0.035
	Yes	No	Yes	No	
Antibiotics	2	9	3	7	0.635
Some breastfeeding	8	3	9	1	0.586
Cat in household	2	9	4	6	0.361
Dog in household	8	3	5	5	0.387

Supplementary Table 2. Cohort demographics with respect to allergic outcomes for samples used for IgA-SEQ subset on the ROC cohort only. Cell values indicate number of infants in each cohort corresponding to each covariate. P-values indicate tests of association using Student's T-Test for continuous variables and Fisher's Exact Test for categorical variables.

SUPPLEMENTARY TABLE 3

Variable	OR	OR CI (lower)	OR CI (upper)	P-value
**deliveryMode = Vaginal Delivery**	0.012	0	0.469	0.011
**Group = ROC**	2.099	0.051	369.127	0.692
**bfStatus = Some human milk**	0.351	0.012	5.246	0.447
**ProbRatio(R.gnavus)**	0	0	0.605	0.034

Supplementary Table 3. Firth Logistic Regression relating IgA-coating of *R. gnavus* to allergic outcomes controlling for lifestyle and population factors.

REFERENCES

1. Jarvinen KM, Davis EC, Bevec E, Jackson CM, Pizzarello C, Catlin E, et al. Biomarkers of Development of Immunity and Allergic Diseases in Farming and Non-farming Lifestyle Infants: Design, Methods and 1 Year Outcomes in the "Zooming in to Old Order Mennonites" Birth Cohort Study. *Front Pediatr*. (2022) 10:916184. doi: 10.3389/fped.2022.916184
2. Clemente JC, Pehrsson EC, Blaser MJ, Sandhu K, Gao Z, Wang B, et al. The microbiome of uncontacted Amerindians. *Sci Adv*. (2015) 1:e1500183.
3. J. Gregory Caporaso CLL, William A. Walters, Donna Berg-Lyons, Catherine A. Lozupone, Peter J. Turnbaugh, Noah Fierer, and Rob Knight. Global patterns of 16S rRNA diversity at a depth of millions of sequences per sample. *Proc Natl Acad Sci U S A*. (2011) 108:4516-4522. doi: 10.1073/pnas.1000080107Downloaded
4. Bolyen E, Rideout JR, Dillon MR, Bokulich NA, Abnet CC, Al-Ghalith GA, et al. Reproducible, interactive, scalable and extensible microbiome data science using QIIME 2. *Nat Biotechnol*. (2019) 37:852-857. doi: 10.1038/s41587-019-0209-9
5. McDonald D, Price MN, Goodrich J, Nawrocki EP, DeSantis TZ, Probst A, et al. An improved Greengenes taxonomy with explicit ranks for ecological and evolutionary analyses of bacteria and archaea. *ISME J*. (2012) 6:610-8. doi: 10.1038/ismej.2011.139
6. Faith DP, and Baker AM. Phylogenetic diversity (PD) and biodiversity conservation: some bioinformatics challenges. *Evolutionary bioinformatics online*. (2006) 2:121-8.
7. Lozupone C, and Knight R. UniFrac: a new phylogenetic method for comparing microbial communities. *Appl Environ Microbiol*. (2005) 71:8228-35. doi: 10.1128/AEM.71.12.8228-8235.2005
8. Jackson MA, Pearson C, Ilott NE, Huus KE, Hegazy AN, Webber J, et al. Accurate identification and quantification of commensal microbiota bound by host immunoglobulins. *Microbiome*. (2021) 9:33. doi: 10.1186/s40168-020-00992-w
9. Firth D. Bias reduction of maximum likelihood estimates. *Biometrika*. (1993) 80:27-38.
10. Georg Heinze MP, Lena Jiricka, Gregor Steiner. logistf: Firth’s Bias-Reduced Logistic Regression. (2025).
11. Campo JJ, Seppo AE, Randall AZ, Pablo J, Hung C, Teng A, et al. Human milk antibodies to global pathogens reveal geographic and interindividual variations in IgA and IgG. *J Clin Invest*. (2024) 134:e168789. doi: 10.1172/JCI168789
12. Huda MN, Ahmad SM, Alam MJ, Khanam A, Kalanetra KM, Taft DH, et al. Bifidobacterium Abundance in Early Infancy and Vaccine Response at 2 Years of Age. *Pediatrics*. (2019) 143. doi: 10.1542/peds.2018-1489
13. Seppo AE, Fridy S, Varrone J, Gill SR, Grier A, Lomas JM, et al. High Microbiome Diversity and IgA Responses in Breast Milk of Old Order Mennonites with a Low Prevalence of Allergic Diseases. *Journal of Allergy and Clinical Immunology*. (2017) 139:AB278. doi: 10.1016/j.jaci.2016.12.893
14. Panigrahi P, Parida S, Nanda NC, Satpathy R, Pradhan L, Chandel DS, et al. A randomized synbiotic trial to prevent sepsis among infants in rural India. *Nature*. (2017) 548:407-412. doi: 10.1038/nature23480

15. Donaldson GP, Ladinsky MS, Yu KB, Sanders JG, Yoo BB, Chou WC, et al. Gut microbiota utilize immunoglobulin A for mucosal colonization. *Science*. (2018) 360:795-800. doi: 10.1126/science.aaq0926
16. Benjamini Y HY. Controlling the false discovery rate: a practical and powerful approach to multiple testing. *Journal of the Royal Statistical Society. Series B (Methodological)*. (1995) 57:289-300.
17. Gu Z, Eils R, and Schlesner M. Complex heatmaps reveal patterns and correlations in multidimensional genomic data. *Bioinformatics*. (2016) 32:2847-9. doi: 10.1093/bioinformatics/btw313
